# Impact of Burnout on Organizational Outcomes, the Influence of Legal Demands: The Case of Ecuadorian Physicians

**DOI:** 10.3389/fpsyg.2018.00662

**Published:** 2018-05-04

**Authors:** Paola Ochoa

**Affiliations:** ESPAE, Graduate School of Management, Escuela Superior Politecnica del Litoral, Guayaquil, Ecuador

**Keywords:** burnout, physicians, outcomes, criminal law, efficacy

## Abstract

Interest in burnout has developed extensively worldwide, but there is scarce the literature regarding the consequences that new legal demands have on burnout and on organizational outcomes in physicians. The global context of the medical profession has been characterized in the recent years by changes in the employment patterns, profound intensification of work, and increment of labor flexibility. In this context, the study aims to analyze the influence of burnout on organizational outcomes in physicians, depending on new legal demands perception in Ecuador. Regarding the method, the research was cross sectional and in the first stage, studied the psychometric characteristics, validity and reliability of the instrument to assess burnout through a series of confirmatory factor analyses (CFA). In a second part, we assessed, the robustness of the model of causal relations between the burnout dimensions and organizational outcomes. We carried out a series of path analysis, structural equation model. The study was accomplished in five hospitals and the sample was incidental, comprising 435 physicians from Ecuador. We divided the group in two subcategories, Sample A, composed by participants that considered that new Criminal Code (COIP) affects them and the Sample B, the group of physicians who believed that the COIP does not affect them. Burnout was assessed with the Spanish adaptation of the Maslach Burnout Inventory (MBI), the Organizational outcomes were measured with a seven-item self-report questionnaire, and we included an item regarding to the influence of new Criminal Code. We formulated four hypotheses, that considered that physicians who believed that the COIP affect them experience a greater negative influence of burnout on organizational outcomes. The results indicated that the group of physicians who believed that the COIP affects them (Sample A) experienced a greater negative influence of cynicism on productivity than Sample B. Moreover, the lack of efficacy dimension had more positive influence on turnover in group that believed that the Criminal Code does not affect their practice. The study is unique because incorporated new legal demands to traditional relation burnout and organizational outcomes in physicians.

## Introduction

The rapid technological changes, the implementation of new management models, the responsibility for the patient's life, the permanence of new working conditions that seem punctual—like instability and temporality—, among many others, have produced an intense labor dynamic that medical professionals must deal with in their daily work.

The global context of the medical profession has been characterized in the recent years by changes in the employment patterns, profound intensification of work, and increment of labor flexibility. Along with these changes in Ecuador, investment in the health sector has increased. Moreover, various conditions of the labor and legal framework have been added, affecting the practice of Ecuadorian physicians. Regarding the labor aspect, through the Organic Law of Public Service (Ministry of Work, 2010), the obligation of the 8-h workday was imposed as of 2010 for civil servants, and contracts for a maximum of 2 years was established.

As a central issue of our study in the social context, we have considered the changes brought about in the legal reality of Ecuador. The Ecuadorian government approved the Organic Comprehensive Criminal Code COIP (Ministry of Justice, 2014) in 2014, which emphasizes the criminal responsibility of professionals, including physicians. The law has generated mixed feelings among those in the medical community of Ecuador. Some of them considered the benefits in order to evaluate medical work, and another group argued about the criminalization of medical practice and limitations on autonomous exercise of the profession. According to Article 146, culpable homicide by professional praxis will be qualified due to the disregard for care practices causing the death of another person (National Cort of Justice, 2014). Within this social and legal framework, we propose the study of the relationship between burnout syndrome and organizational outcomes considering the influence of the new law (COIP).

An interest in burnout syndrome has extensively increased worldwide and currently comprises a set of literature with over 6,000 books, chapters, and articles (Schaufeli et al., 2009). The research on burnout includes samples of different occupations such as teachers (e.g., Nizielski et al., 2013; Tijdink et al., 2013), civil servants (e.g., Rivera et al., 2015), and health professionals, as in the present study (e.g., Bragard et al., 2014).

In this study, burnout is considered a response to chronic job stress constituting attitudes and negative feelings toward people with whom one works and toward one's own professional role, and is primarily characterized by the experience of feeling physically and emotionally exhausted (Maslach et al., 2001; Gil-Monte and Moreno, 2007). The burnout syndrome has three dimensions: exhaustion, cynicism, and a lack of professional efficacy (Bresó et al., 2007).

An analysis of the influence of the burnout syndrome on organizational outcomes was carried out within the theoretical framework of the demands-resources model (Bakker and Demerouti, 2007). The balance between demands and resources can generate two types of consequences for the employee. On the one hand, there are positive consequences leading to psychological well-being and work engagement, and on the other hand, there are negative consequences, which can lead to stress, burnout, and other diseases.

Since the initial studies of burnout (Maslach and Jackson, 1981; Maslach, 2001), there has been a great interest in confirming, testing, and discovering how burnout impacts employee performance and organizational outcomes. Numerous studies have examined the relationship between burnout and outcomes (e.g., Lee and Ashforth, 1996; Melchior et al., 1997); however, according to Swider and Zimmerman (2010) in their meta-analysis on burnout and work outcomes, there are around 20 studies that have focused on each dimension of burnout and job performance. In this sense, the effects of burnout on patient management (e.g., Berland et al., 2008), the quality of service (e.g., Klein et al., 2010; Faller et al., 2011), and turnover intention (e.g., Van Bogaert et al., 2010, 2013) have been investigated.

These results include the human, organizational, and socio-economic facets. From the human point of view, malaise and psychosocial risks have many consequences, among which are depersonalization, decline of the doctor-patient relationship, and a sense of professional frustration. According to the ethics of patient care (Gandi et al., 2011), quality health care must include not only the process of medical attention, but also that of caring, personal attention, and knowledge of the patient's identity.

From the organizational perspective, factors such as work overload, the demands of administrative tasks combined with professional tasks, ethical tensions to meet the Hippocratic requirements, and business goals become psychosocial risk factors, leading to a decline in the quality of health care (e.g., Humphries et al., 2014), and the lack of attention to the care of patients (e.g., Montgomery et al., 2013) with serious consequences to the patient, health services, and the society as a whole.

From the socio-economic viewpoint, according to Joffre-Velázquez et al. (2008), burnout is the source of 50% of the sick leaves in the European Union and annually affects up to 40 million workers, which costs more than 20 million euros. Studies carried out in the United States (Burke, 2014) have also shown high costs due to work absenteeism caused by stress-related conditions and costs due to diseases.

**Figure 1:**
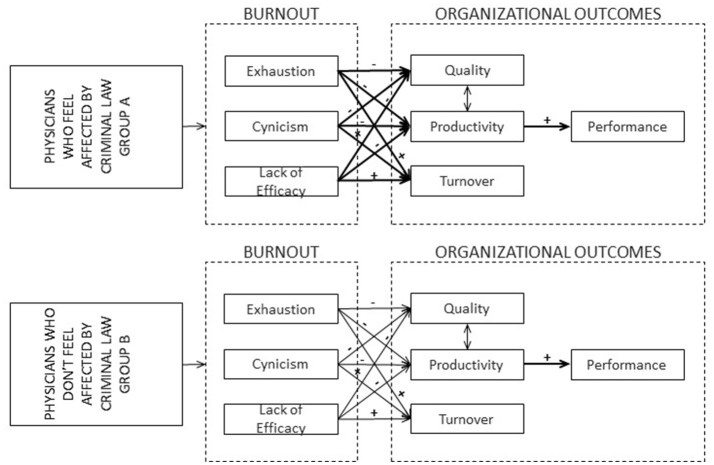
Theoretical model of impact of burnout on organizational outcomes, the influence of legal demands: the case of Ecuadorian physicians.

From the proposed theoretical framework, we hypothesized a causal model to analyze the impact of burnout syndrome on organizational outcomes in two groups of physicians; one which thinks that the COIP negatively affects their job and other that considers that the COIP does not affect their job (see Figure 1). This approach was broken down into a series of hypotheses which are as follows:

*Hypothesis 1*. The group of physicians who perceive that the COIP affects them (Sample A) will experience a greater negative influence of the exhaustion and cynicism dimensions on organizational results (quality, productivity) than the group that believe that the law does not affect them (Sample B).
*Hypothesis 2*. The group of physicians who perceive that the COIP affects them (Sample A) will experience a greater influence of exhaustion and cynicism dimensions on turnover than the group that believes that the law does not affect them (Sample B).
*Hypothesis 3*. The group of physicians who believes that the COIP affects them (Sample A) will experience a greater negative influence of the lack of efficacy on quality and productivity than the group that believes that the COIP does not affect them (Sample B).
*Hypothesis 4*. The group of physicians who believe that the COIP affects them (Sample A) will experience a greater negative influence of the lack of efficacy on turnover than the group that believes that the law does not influence them (Sample B).

## Methods

The study sample was incidental, comprising 435 physicians from the city of Guayaquil. With respect to gender, the majority were men (58%). In relation to age, the majority was the group between 35 and 45 years (37.7%), followed by the group between 24 and 34 years (32.3%), the group between 35 and 46 years (21.8%), and the group aged 57 years and above (8.2%). Concerning to the type of contract, 73.65% had a temporary contract and 26.4% had a permanent bond. As regards to the supervisory responsibilities, sample showed 58% of respondents without supervisory responsibilities and 42% with them. The distribution by department was 67.6% were in surgical medical departments, 28.1% in critical care departments, and 4.4% in administrative units. With regard to their work contract, 94.5% worked full-time, 4.85% worked part-time, and 0.7% other.

Burnout was assessed using the Spanish adaptation of the Maslach Burnout Inventory (MBI) developed by Bresó et al. (2007). The MBI has 15 items that rate the burnout dimensions on a seven-point Likert scale ranging from 0 (never) to 6 (always). Exhaustion was measured with five items (e.g., “I feel burned out from my work,” and “Working all day is really a strain for me.”). Cynicism was measured with five items; (e.g., “I have become less enthusiastic about my work,” and “I have become more cynical about whether my work contributes anything.”). Efficacy was measured with six items (e.g., “In my opinion, I am good at my job” and “I feel exhilarated when I accomplish something at work.”) For efficacy dimension, we measured the lack of efficacy with the corresponding reversed efficacy scale (Schaufeli and Salanova, 2007).

Organizational outcomes were measured using a seven-item self-report questionnaire. All items were rated on a seven-point Likert scale to assess different organizational outcomes: turnover, quality, productivity, and performance. Turnover intention was measured with the three items from Mobley's scale (Mobley et al., 1978) as follows: “In the last few months have you ever thought seriously about looking for a job,” “In the last few months have you ever thought seriously about looking for a job?” and “As soon as possible I'm going to abandon the organization.” The employees rated the quality of their work with one item “The quality of my work is…” and productivity was measured with two items: “ I complete my work on time,” and “The productivity of my performance in my work is…” Lastly, employees reported the result of the previous organizational assessment of their performance using the item, “According to the last report of the organization where you work, what was the result of your last performance evaluation?” The reliability of the scales was assessed by the Cronbach's alpha index and the composite reliability (rho index) in the framework of the confirmatory factor analysis. The analysis of reliability for each measure showed good levels of Cronbach's alpha (Exhaustion α = 0.918; Cynicism α = 0.845; lack of efficacy α = 0.855; Turnover α = 0.848; Productivity α = 0.802). The Cronbach's alphas for quality and performance variables were not reported because they included only one item in their scales. Further, to examine the perception of the consequences of the new COIP, we included an item about the influence of the new COIP, which measures how the COIP affect physicians. We classified the answers into two samples: Sample A comprising 54.4% of the participants who believe that the COIP affects them and Sample B comprising 32% participants who believe that the COIP does not negatively affect their practice. The rest of the group−15.6% of the physicians—were indifferent to the law, and we did not include them in the analysis.

This study was approved by the Ethics Commission of ESPOL ESPAE. In its development, the international rules regarding written informed consent, scientific treatment of the data, confidentiality of data of participants and institutions, the anonymity of the answers, and scientific and responsible use of the information were followed. After obtaining the data, the analysis was conducted in two phases. In the first stage, we studied the psychometric characteristics, validity, and reliability of the instrument used to assess burnout through a series of confirmatory factor analyses (CFA). Given that the burnout dimensions were assessed using Likert-type scales, the fit of several hypothetical models to the structure of the questionnaire was assessed with robust unweighted least squares estimation method (McDonald, 1982). To estimate the fit between the sample variance-covariance matrices and the matrix generated by the hypothesized models to the factor structure of the instrument, we used the following indexes: the scaled Satorra and Bentler (1994) index ($\chi_{\text{S}-\text{B}}^{2}$), the root mean square error of approximation (RMSEA) (Steiger, 1990), the non-normed fit index (NNFI) (Bentler and Bonett, 1980), and the comparative fit index (CFI) (McDonald and Marsh, 1990). On the basis of the results obtained in the CFAs, we estimated reliability with the composite reliability index (Raykov, 1997).

In the second stage, we assessed—among the two groups of physicians—the robustness of the model of causal relationships among the burnout dimensions: exhaustion, cynicism, lack of efficacy, and organizational outcomes—quality, productivity, and intention of quitting—derived from the formulated hypotheses. We carried out a series of path analysis and structural equation modeling with the observed variables. Due to the small samples size of the present study, a model with only observed variables was preferred over one with latent variables. After analyzing the fit of the model of both samples we analyzed the direct, indirect, and total effects of burnout syndrome on organizational outcomes.

We then studied the relationships among the burnout dimensions and the organizational outcomes that configure the structural model. Statistical softwares IBM-SPSS 22 and LISREL 8.80 (Jöreskog and Sörbom, 2006) were used for data analysis.

## Results

The results are presented in three parts. First, we explain the results corresponding to the analyses of the validity and reliability of the burnout inventory. Second, we present the descriptive information, and third, we present the results of the path analysis.

The construct validity of burnout was analyzed by testing the fit of three hypotheses about its factor structure: a one-dimensional model, a three-orthogonal-factors model, and an oblique three-dimensional model. The one-dimensional model that comprised all the items of the scale clearly indicated an insufficient fit ($\chi_{\text{S}-\text{B}}^{2}$ = 2935.712, df = 90, RMSEA = 0.272, NNFI = 0.632, CFI = 0.685). Similarly, the Hypothesis of a model consisting of three orthogonal factors corresponding to the dimensions of exhaustion, lack of efficacy, and cynicism did not show an adequate fit between the sample matrix and the matrix generated by the model (${\chi \;}_{\text{S}-\text{B}}^{2}$ = 485.576, df = 93, RMSEA = 0.099, NNFI = 0.927, CFI = 0.937). Finally, the results showed that the oblique three-dimensional model demonstrated the best fit between the sample variance-covariance matrix and the matrix generated by the restrictions imposed on the model. All the fit indices were above the cut-off points that are indicative of a good fit ($\chi_{\text{S}-\text{B}}^{2}$ = 274.934, df = 87, RMSEA = 0.071, NNFI = 0.963, CFI = 0.969). However, a detailed examination of the obtained lambda parameters showed that item 3 of Cynicism had a minimum factorial weight; therefore, we proceeded to redefine the factorial model, eliminating this item. The results obtained in the CFA of the model made up of three oblique dimensions—exhaustion, cynicism, and lack of efficacy—without item 3 of the cynicism dimension showed a good fit (${\chi \;}_{\text{S}-\text{B}}^{2}$ = 209.901, df = 74, RMSEA = 0.065, NNFI = 0.972, CFI = 0.977). Table 1 presents the fully standardized solution of the model obtained. In this table, it can be seen that for the Exhaustion dimension, the factor loadings were homogeneous, ranging between 0.879 and 0.804, and items 5 and 3 had the highest factor loadings. As for the Cynicism dimension, the items also showed homogeneous and high values ranging between 0.860 and 0.717, with item 1 presenting the highest loading at 0.860, and item 4 the lowest at 0.717. Concerning the lack of Efficacy dimension, the items presented more heterogeneous weights, ranging from 0.825 to 0.560, which correspond to items 5 and 1, respectively.

**Table 1:** Standardized lambda parameters of the three oblique dimension model of the factorial structure of burnout.

	**Exhaustion**	**Cynicism**	**Lack of efficacy**
Exhaus1	0.836	–	–
Exhaus2	0.811	–	–
Exhaus3	0.853	–	–
Exhaus4	0.804	–	–
Exhaus5	0.879	–	–
Cynicis1	–	0.860	–
Cynicis2	–	0.843	–
Cynicis4	–	0.717	–
L-Effica1	–	–	0.560
L-Effica2	–	–	0.677
L-Effica3	–	–	0.785
L-Effica4	–	–	0.754
L-Effica5	–	–	0.825
L-Effica6	–	–	0.597

*All parameters are statistically significant (p < 0.001)*.

In addition to examining the construct validity of the scale, we assessed its reliability with the rho composite reliability index obtained from the CFA (Raykov, 1997). The results showed high levels of reliability for all three dimensions: Exhaustion ρ = 0.922; Cynicism ρ = 0.850, and lack of Efficacy ρ = 0.854.

Since the study data were obtained from the same source—the employees—and through self-reports, it was necessary to evaluate the presence of bias due to the common method variance. For this purpose and in accordance with the proposal of Podsakoff et al. (2003), we conducted a series of CFA to assess and compare the fit between a measurement model consisting of seven oblique factors—corresponding to the three burnout dimensions and the four dimensions of the organizational results—and a measurement model comprising these seven oblique factors and one factor method which included all items. The results showed a good fit of the model consisting of the seven dimensions (${\chi \;}_{\text{S}-\text{B}}^{2}$ = 578.589, df = 181, RMSEA = 0.073, NNFI = 0.955, CFI = 0.961). Incorporating the factor method led to a slight improvement in the fit (${\chi \;}_{\text{S}-\text{B}}^{2}$ = 347.542, df = 160, RMSEA = 0.054, NNFI = 0.976, CFI = 0.982); therefore, the presence of a bias associated with the common method variance cannot be fully eliminated (*TR*_d_ = 360,062; Δgl = 21; *p* < 0.000; ΔRMSEA = −0.019; ΔNNFI = 0.021; ΔCFI = 0.021; Satorra and Bentler, 1994).

The means, standard deviations, and correlations among the study variables for the two groups of physicians are presented in Table 2. The table shows the means, standard deviations, and correlations among the study variables in the group of physicians who believe that the COIP negatively affects them (hereinafter Sample A, N_A_ = 228). In this group, Exhaustion was negatively related to quality (*r* = −0.17, *p* < 0.01), productivity (*r* = −0.20, *p* < 0.01), and performance (*r* = −0.21, *p* < 0.01); it was positively related to turnover (*r* = 0.42, *p* < 0.01). Cynicism was negatively related to quality (*r* = −0.42, *p* < 0.01), productivity (*r* = −0.46, *p* < 0.01), and performance (*r* = −0.34, *p* < 0.01), and positively related turnover (*r* = 0.31, *p* < 0.01). Efficacy was negatively related to turnover (*r* = −0.12, *p* < 0.01) and positively related to quality (*r* = 0.62, *p* < 0.01), productivity (*r* = 0.63, *p* < 0.01), and performance (*r* = 0.50, *p* < 0.01).

**Table 2:** Means, standard deviations, and correlations between the variables in Samples A and B.

	***M***	***SD***	**1**	**2**	**3**	**4**	**5**	**6**	**7**
**SAMPLE A (*N* = 228)**									
1. Exhaustion	3.05	1.66							
2. Cynicism	2.67	1.44	0.544^*^						
3. Lack of Efficacy	5.02	0.92	−0.085	−0.355^*^					
4. Quality	5.95	1.38	−0.174^*^	−0.428^*^	0.623^*^				
5. Productivity	5.89	1.31	−0.203^*^	−0.466^*^	0.631^*^	0.885^*^			
6. Turnover	3.43	1.85	0.423^*^	0.317^*^	−0.122	−0.056	−0.110		
7. Performance	6.15	1.31	−0.214^*^	−0.348^*^	0.505^*^	0.676^*^	0.655^*^	−0.126	
**SAMPLE B (*N* = 139)**									
1. Exhaustion	2.14	1.73							
2. Cynicism	2.00	1.45	0.662^*^						
3. Lack of Efficacy	5.00	1.27	0.725	0.902					
4. Quality	6.56	0.80	−0.206^*^	−0.340^*^	0.271^*^				
5. Productivity	6.52	0.84	−0.213^*^	−0.324^*^	0.195^*^	0.775^*^			
6. Turnover	3.19	2.15	0.433^*^	0.253^*^	−0.206^*^	−0.161^*^	−0.153^*^		
7. Performance	6.38	1.06	−0.253^*^	−0.265^*^	0.239^*^	0.610^*^	0.613^*^	−0.143	

M, Means; SD, standard deviations, and

* *p < 0.01*.

Table 2 shows the means, standard deviations, and correlations between the study variables in the group of physicians who believe that the COIP does not affect them (hereinafter Sample B, N_B_ = 139). In this group, Exhaustion was negatively related to quality (*r* = −0.20, *p* < 0.01), productivity (*r* = −0.21, *p* < 0.01), and performance (*r* = −0.25, *p* < 0.01); and positively related to turnover (*r* = 0.43, *p* < 0.01). Cynicism was negatively related to quality (*r* = −0.34, *p* < 0.01), productivity (*r* = −0.32, *p* < 0.01), and performance (*r* = −0.26, *p* < 0.01), and positively related to turnover (*r* = 0.25, *p* < 0.01). Efficacy was negatively related to turnover (*r* = −0.20, *p* < 0.01) and positively related to quality (*r* = 0.27, *p* < 0.01), productivity (*r* = 0.19, *p* < 0.01), and performance (*r* = 0.23, *p* < 0.01).

For a global assessment of the impact of burnout on organizational results based on the perception of medical practice law, we carried out a series of path analyses of the model of the hypothesized causal relationships (see Figure 1). According to this model, the dimensions of burnout—Exhaustion, Cynicism, and lack of Efficacy—are exogenous variables and predictors, and organizational outcomes such as quality, productivity, and turnover are endogenous variables. We carried out a structural equations modeling using the method of path analysis; we tested the adjustment of the two samples.

Regarding Sample A—comprising employees who perceive that regulatory changes affect their medical practice—the results showed an appropriate adjustment of the hypothesized model. In this sense, all the considered indices had values above the cut-offs that indicate a good fit of the model evaluated. The value obtained in the scaled chi-square Satorra-Bentler (χ^2^ = 10.611, gl = 6, *p* = 0.101) allows to accept equality between the sample variance-covariance matrix and the generated from the hypothesized model. Similarly, the root mean square error of approximation (RMSEA = 0.0597, CI 90% = 0.000–0.117), the non-normed fit index (NNFI = 0.983), and the Comparative Fit Index (CFI = 0.995) had values well above the cut-offs that are considered necessary for a good fit of the model.

Table 3 shows the standardized coefficients of the parameters of the model corresponding to both the direct and indirect effects and the total effects. Regarding the direct effects of the dimensions of burnout on organizational results, exhaustion primarily affected the rotation [γ_(3, 1)_ = 0.384, *p* < 0.05], while cynicism [γ_(2, 2)_ = −0.367, *p* < 0.05] and lack of efficacy [γ_(2, 3)_ = −0.469, *p* < 0.05] affected productivity although in a reverse mode. Cynicism and lack of efficacy negatively affected the productivity. The rest of the gamma parameters corresponded to the direct effects of burnout on organizational outcomes (quality, productivity, and turnover) and had a very small weight, which was also not significant.

**Table 3:** Standardized direct effects, indirect effects, and total effects.

	**Sample A**			**Sample B**		
	**Exhaustion ↓**	**Cynicism ↓**	**Lack of efficacy ↓**	**Exhaustion ↓**	**Cynicism ↓**	**Lack of efficacy ↓**
**DIRECT EFFECTS**						
Quality	0.021	−0.010	−0.118	0.043	−0.089	−0.128
Productivity	0.043	−0.367^*^	−0.469^*^	0.017	−0.331^*^	−0.144
Turnover	0.384^*^	0.073	0.046	0.389^*^	0.059	0.191^*^
**INDIRECT EFFECTS**						
Quality	0.033	−0.283^*^	−0.362^*^	0.011	−0.222^*^	−0.097
Performance	0.035	−0.233^*^	−0.337^*^	0.016	−0.215^*^	−0.107
**TOTAL EFFECTS**						
Quality	0.054	−0.293^*^	−0.481^*^	0.054	−0.311^*^	−0.225
Productivity	0.043	−0.367^*^	−0.469^*^	0.017	−0.331^*^	−0.144
Performance	0.035	−0.233^*^	−0.337^*^	0.016	−0.215^*^	−0.107
Turnover	0.384^*^	0.073	0.046	0.389^*^	0.059	0.191^*^

* *p < 0.05*.

**Figure 2:**
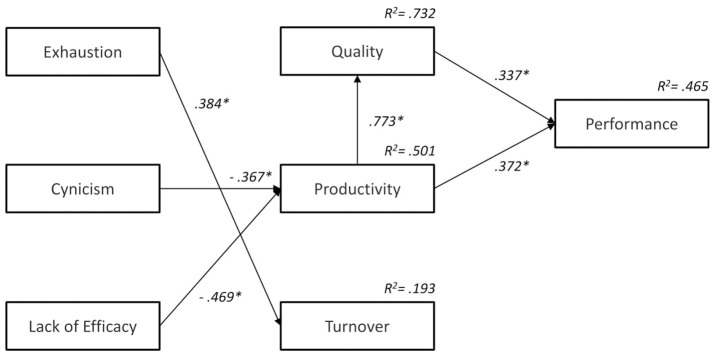
Path Analysis Results of Physicians who believe that the medical malpractice law affects negatively them (Sample A). We eliminated not significant parameters. Standardized coefficients and *R*^2^-values of endogenous variables. ^*^p < 0.05.

Concerning the indirect effects on quality and performance, the influence on the dimensions of burnout was very different. Before analyzing these indirect effects, we examine the mediating role played by relationships among the results productivity, quality, and performance (see Figure 2). The results confirmed the influence of productivity on quality [β_(1, 2)_ = 0.773, *p* < 0.05], productivity and the quality on performance [β_(4, 2)_ = 0.372, *p* < 0.05; β_(4, 1)_ = 0.337, *p* < 0.05]. These relationships had a mediating role between the dimensions of burnout and the quality and performance. As shown in Table 3, exhaustion did not significantly affect quality or performance. On the contrary, both cynicism and the lack of efficacy had an impact on the quality and performance. Cynicism negatively influenced both quality [γ_(2, 2)_ β_(1, 2)_ = −0.283, *p* < 0.05] and performance [γ_(2, 2)_ β_(1, 2)_ β_(4, 1)_ = −0.233, *p* < 0.05], and the lack of efficacy had a negative impact on the quality [γ_(2, 3)_ β_(1, 2)_ = −0.362, *p* < 0.05] and performance [γ_(2, 3)_ β_(4, 2)_ = −0.337, *p* < 0.05]. Finally, as indicated in Table 3, the effects of the dimensions of burnout on the organizational results of Sample A were very different for each dimension. Exhaustion moderately influencing turnover was the only dimension of burnout that does it in the sample. Cynicism negatively and moderately affected quality, productivity, and performance. Finally, the lack of efficacy was the dimension in Sample A that had the greatest impact on the results of quality, productivity, and performance.

Regarding Sample B—comprising physicians who believe that regulatory changes do not affect their medical practice—the results showed an appropriate adjustment of hypothesized model. In this sense, all the considered indices had values above the cut-offs that indicate a good fit of the model evaluated. The value obtained in the scaled chi-square Satorra-Bentler (χ^2^ = 8.656, gl = 6, *p* = 0.194) allows to accept the equality between the sample variance-covariance matrix and the matrix generated from the hypothesized model. Similarly, the root mean square error of approximation (RMSEA = 0.0586, CI 90% = 0.000–0.138), the non-normed fit index (NNFI = 0.972) and the Comparative Fit Index (CFI = 0.992) had values well above the cut-offs that are considered necessary for a good fit of the model.

Table 3 shows the standardized coefficients of the parameters of the model corresponding to both the direct and indirect effects and the total effects. Regarding the direct effects of the dimensions of burnout on organizational results, exhaustion primarily affected turnover [γ_(3, 1)_ = 0.389, *p* < 0.05], while cynicism negatively affected productivity [γ_(2, 2)_ = −0.331, *p* < 0.05], and lack of efficacy positively affected turnover [γ_(3, 3)_ = 0.191, *p* < 0.05]. The rest of the gamma parameters corresponded to the direct effects of burnout on organizational outcomes (quality, productivity, and turnover) and had a very small weight, which was also not significant.

**Figure 3:**
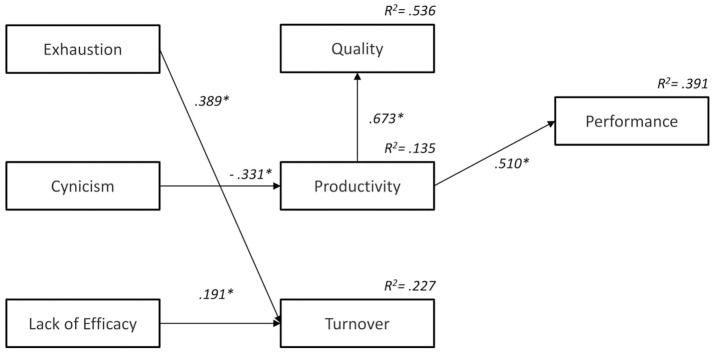
Path Analysis Results of Physicians who believe that medical malpractice law doesn't influence them (Sample B). We eliminated not significant parameters. Standardized coefficients and *R*^2^ values of endogenous variables. ^*^p < 0.05.

Regarding the indirect effects on quality and performance, the influence of the dimensions of burnout was different in each case. Before analyzing these indirect effects, we examine the mediating role played by relationships among the results productivity, quality, and performance (see Figure 3). The results confirmed the influence of productivity on quality [β_(1, 2)_ = 0.673, *p* < 0.05] and performance [β_(4, 2)_ = 0.510, *p* < 0.05]. As shown in Table 3, exhaustion and the lack of efficacy did not significantly affect quality or performance. Only cynicism had a significant impact on quality [γ_(2, 2)_ β_(1, 2)_ = −0.222, *p* < 0.05] and performance [γ_(2, 2)_ β_(4, 2)_ = −0.215, *p* < 0.05]. Concerning total effects, the lack of efficacy and exhaustion had a positive impact on turnover. Cynicism negatively affected quality, productivity, and performance. Finally, as indicated in Table 3, the effects of the dimensions of burnout on the organizational results in Sample B were very different for each dimension. Exhaustion influenced turnover, cynicism negatively affected quality, productivity, and performance, and finally, the lack of efficacy had a positive influence on turnover.

In sum, the Hypothesis 1 was confirmed in part because in Sample A, cynicism had a greater influence on productivity than in Sample B. However, the influence of exhaustion on quality and productivity was not significant in Sample A or Sample B. Hypothesis 2 was not confirmed because in Sample A exhaustion did not have a greater influence on turnover than in Sample B, and the influence of cynicism on turnover was not significant.

Furthermore, Hypothesis 3 was not confirmed because in Sample A, the lack of efficacy had a negative influence on productivity; however, it did not have a significant effect on quality, and in Sample B, the lack of efficacy did not significantly affect quality and productivity. Finally, Hypothesis 4 was not confirmed because the lack of efficacy did not have a greater negative influence on the turnover in Sample A than in Sample B. The results showed that the lack of efficacy had a positive influence on turnover in Sample B (comprising physicians who believe that regulatory changes do not affect their medical practice), and it was not significant in Sample A.

## Discussion

The cognitive, emotional, and physical demands of doctors' job require a great effort in their work. If this requirement is not counterbalanced with employee resources and organizational resources (technological resources, social environment, and organizational assets; Bakker and Demerouti, 2007), it can lead to a loss of meaning of work (Blanch et al., 2012), lack of commitment, and more influence of psychosocial risks.

We highlight two elements when considering the results obtained in the causal model of burnout and organizational outcomes, influenced by the perceptions toward the COIP, in Ecuador. First, the distinction made for the relationships among each burnout dimension and each organizational outcome by the groups of physicians let observe relevant and significant common relationships in both groups, the positive influence of exhaustion on turnover, and the negative influence of cynicism on productivity. Second, the inclusion of the socio-legal variable (the perception toward the COIP) in our model of burnout-organizational outcomes brought a new evidence of influence of socio-legal perceptions on burnout and organizational results.

Our study has some similarities and differences with Swider and Zimmerman (2010) meta-analysis' results. The pattern of correlations among the dimensions of burnout and turnover is similar to that obtained in Swider and Zimmerman (2010). However, the intensity of the correlation among the burnout dimensions and turnover was greater in our study. The correlations among the dimensions of burnout and job performance also confirm the results obtained by Swider and Zimmerman (2010) regarding the pattern of the relationship. In this case, the intensity of the differences was minor; the values obtained were always within the confidence intervals to 95% of the true correlation seen in Swider and Zimmerman (2010) study. For example, the correlation between efficiency and performance in our study had the same intensity as in Swider and Zimmerman (2010) meta-analysis. In summary, our research sought to address one of the conclusions of the work of Swider and Zimmerman (2010), the need for further analysis of the relationship among the dimensions of burnout and organizational results, based on the legal variable that we used.

In highly demanding emotional and labor services such as medical practice, studying the negative impact of the burnout syndrome on employment outcomes such as quality (Kanai-Pak et al., 2008; Klein et al., 2010; Poghosyan et al., 2010) should be an essential concern of the professionals, administrative staff, and human resources managers. One of the objectives of the current research was to examine the impact of exhaustion and cynicism on the working dynamic and delivery service of physicians, as well as the status of the decline of the physician-patient relationship. These consequences play an important role in the escalation of severe social effects that affect the doctor-patient social contract.

The results showed a negative relationship between the cynicism and productivity as reported in other studies that globally assessed burnout and its influence on productivity (e.g., Kim et al., 2013; Dewa et al., 2014). The separate analysis of the relationship between burnout—taking each of its dimensions independently—and productivity can indicate different impacts of dimensions and provide evidence of the advantage of differentiating the construct in the analysis of the burnout-organizational outcomes. Through our differentiated analysis, the study compared the groups of physicians, but the influence of exhaustion on quality and productivity was not significant in Sample A or Sample B (Hypothesis 1). This result on the quality and productivity is not consonant with the influence of negative legal pressure on the work outcomes and physicians' experience according to other studies (Mello et al., 2005).

Exhaustion and turnover intention showed a positive relationship in Sample A, which supports and confirms both the results obtained in the previous research with global burnout and turnover indices (Faller et al., 2011; Zhang and Feng, 2011) as well as the results obtained in studies that specifically examined the influence of exhaustion on turnover intention (Van Bogaert et al., 2010; Huynh et al., 2014). We noted the particularly important findings concerning the influence of exhaustion on outcomes as this dimension is the most visible and documented of all the burnout dimensions, but the analysis of its consequences is restricted to the personal sphere. The results of this research extend on the study of the consequences of exhaustion to the organizational sphere and found that in this case, the Hypothesis 2 was not confirmed because in Sample A, exhaustion did not have a greater influence on turnover than in Sample B, and the relationship between cynicism and turnover was not significant.

With regard to other relationships that can be derived from the model but are not part of its core, such as the links between productivity and organizational performance, the results of the present study confirmed their relationship (Bellou and Andronikidis, 2008). However, the association among productivity and performance within the causal model related to the burnout dimensions and organizational outcomes would open doors to continue the assessment of joint linkages and direct and indirect relationships in the burnout-outcome models.

Concerning the influence of the perceptions toward the COIP on burnout, physicians who believed that the law negatively affects them did not show differences in terms of the intensity of the relationship between exhaustion and organizational results. However, the results indicated that the group of physicians who felt that they were negatively affected by the criminal code experienced a greater negative influence of cynicism on productivity than physicians who believed that the criminal code does not affect their practice. Factors such as pressure and stress regarding the potential legal demands on doctors, greater control over their daily practice, decline in the meaning of medical profession, uncertainty about how the COIP will be operationalized are the possible explanations for higher depersonalization and lesser productivity. Regarding the influence of law at work according to Mello et al. (2005), malpractice reforms and legal pressures affect the supply of specialist physicians in some areas, may change the physician' scope of practice, and affect patients' access to care. From the legal and social perspectives, the liability reforms (Mello et al., 2005; Frakes, 2015) modify the way by which physicians are evaluated by the society. All these factors should be considered for a global analysis of the legal variables and their influence on organizational results.

The study especially confirms the significant implications of self-efficacy (Bandura, 2012) in the achievement of organizational goals. On the one hand group of physicians who felt that they are negatively affected by the COIP emphasized the negative influence of the lack of professional efficacy on productivity and the indirect influence on quality and performance. It means that Hypothesis 3 was not confirmed totally because in Sample A, the lack of efficacy did not affect the quality significantly, and it even had a negative influence on productivity. In Sample B, the lack of efficacy did not affect the quality and productivity significantly. The contextual, legal, and social demands certainly influenced the lack of efficacy on outcomes such as productivity because physicians feel stressed and uncertain to assume the responsibilities of practice, which could reduce their productivity in terms of the numbers of operations, consultancies, and patients, the quality and characteristics of their service, and in conclusion their performance. Therefore, it is seen that new demands such as legal requirements (COIP) are superior to their personal and professional resources (Bakker and Demerouti, 2007).

On the other hand, regarding the influence of the lack of efficacy on turnover, Hypothesis 4—that the group of physicians who believed that the COIP affects them would experience a greater negative influence of the lack of efficacy on turnover than the group that believed that the law did not influence them—was not confirmed. The results showed that the lack of efficacy had a positive influence on turnover only among physicians who perceived that legal demands do not affect their medical practice. The relationship between the lack of efficacy and organizational outcomes is not lineal and required a multiple perspective of analysis in our study. According to the results, the physicians who perceived that the regulatory changes do not affect their medical practice get the impulse to leave the organization due to the lack of efficacy. The internal locus of control and the personal control of physicians (Bandura and Schunk, 1981) are definitely not enough to cope with other organizational and social changes and physicians consider leaving the organization. Although they try to be resilient, the reserves of their psychological capital (Luthans et al., 2008) comprising hope, resilience, optimism, and efficacy are not enough. In sum, the results of the professional efficacy of this group permit to say that the tensions produced not only by organizational and social demands but also the new law affect the lack of efficacy, the opposite of efficacy, a key individual resource (Bakker and Demerouti, 2007).

Interconnected with the idea of including the perception toward COIP in the model of the burnout-organizational outcomes, this aspect has implications for taking into account contextual variables. Contextual and social variables highly influence physicians' practice, meaning of working, and well-being (Haller and Hadler, 2006; Ochoa and Blanch, 2016). We enriched our model with this legal requirement, a new social demand, and tried to draw attention to the new model of burnout-organizational outcomes, which integrated the complexity of social and legal variables (Wasti et al., 2016).

The limitations of the study are the difficulties to apply it to other contexts because it is based on the situation of Ecuador, the use of an incidental sample, and self-reports scales. The main strength of this research is that it incorporated a different relationship to the many studies that focused on burnout and organizational outcomes (e.g., Lee and Ashforth, 1996; Melchior et al., 1997; Swider and Zimmerman, 2010) as it was added to the model on burnout and organizational results, which is an element of the legal context of the country. Adding the variable—law of medical malpractice “COIP”—also has a real and social impact on the medical profession because of the implications that its application has in terms of greater control over the exercise of the profession, increased emotional demands, and work pressure. In sum, we carried out a particular and unique study by incorporating new contextual demands in the well-known burnout and organizational outcomes and job demands and resources models (Bakker and Demerouti, 2007). This permitted us to observe a new socio-legal dynamic among physicians at work, which surprised us with the results.

## Author contributions

PO was in charge of the study's overall design and carried out the study, including the processing of data, performing the results and analysis.

### Conflict of interest statement

The author declares that the research was conducted in the absence of any commercial or financial relationships that could be construed as a potential conflict of interest.
